# The Effects of Tinnitus in Probabilistic Learning Tasks: Protocol for an Ecological Momentary Assessment Study

**DOI:** 10.2196/36583

**Published:** 2022-11-11

**Authors:** Lili Zhang, Greta Monacelli, Himanshu Vashisht, Winfried Schlee, Berthold Langguth, Tomas Ward

**Affiliations:** 1 Insight Science Foundation Ireland Research Centre for Data Analytics Dublin City University Dublin Ireland; 2 In The Wild Research Limited Dublin Ireland; 3 Department of Psychiatry and Psychotherapy University Regensburg Regensburg Germany

**Keywords:** chronic tinnitus, computational modeling, decision-making, ecological momentary assessment, mobile phone

## Abstract

**Background:**

Chronic tinnitus is an increasing worldwide health concern, causing a significant burden to the health care system each year. The COVID-19 pandemic has seen a further increase in reported cases. For people with tinnitus, symptoms are exacerbated because of social isolation and the elevated levels of anxiety and depression caused by quarantines and lockdowns. Although it has been reported that patients with tinnitus can experience changes in cognitive capabilities, changes in adaptive learning via decision-making tasks for people with tinnitus have not yet been investigated.

**Objective:**

In this study, we aim to assess state- and trait-related impairments in adaptive learning ability on probabilistic learning tasks among people with tinnitus. Given that performance in such tasks can be quantified through computational modeling methods using a small set of neural-informed model parameters, such approaches are promising in terms of the assessment of tinnitus severity. We will first examine baseline differences in the characterization of decision-making under uncertainty between healthy individuals and people with tinnitus in terms of differences in the parameters of computational models in a cross-sectional experiment. We will also investigate whether these computational markers, which capture characteristics of decision-making, can be used to understand the cognitive impact of tinnitus symptom fluctuations through a longitudinal experimental design.

**Methods:**

We have developed a mobile app, AthenaCX, to deliver e-consent and baseline tinnitus and psychological assessments as well as regular ecological momentary assessments (EMAs) of perceived tinnitus loudness and a web-based aversive version of a probabilistic decision-making task, which can be triggered based on the participants’ responses to the EMA surveys. Computational models will be developed to fit participants’ choice data in the task, and cognitive parameters will be estimated to characterize participants’ current ability to adapt learning to the change of the simulated environment at each session when the task is triggered. Linear regression analysis will be conducted to evaluate the impacts of baseline tinnitus severity on adapting decision-making performance. Repeated measures linear regression analysis will be used to examine model-derived parameters of decision-making in measuring real-time perceived tinnitus loudness fluctuations.

**Results:**

Ethics approval was received in December 2020 from Dublin City University (DCUREC/2021/070). The implementation of the experiments, including both the surveys and the web-based decision-making task, has been prepared. Recruitment flyers have been shared with audiologists, and a video instruction has been created to illustrate to the participants how to participate in the experiment. We expect to finish data collection over 12 months and complete data analysis 6 months after this. The results are expected to be published in December 2023.

**Conclusions:**

We believe that EMA with context-aware triggering can facilitate a deeper understanding of the effects of tinnitus symptom severity upon decision-making processes as measured outside of the laboratory.

**International Registered Report Identifier (IRRID):**

PRR1-10.2196/36583

## Introduction

### Background

Tinnitus is an increasingly significant health concern characterized by the perception of sound in the absence of external stimuli [1]. Tinnitus is normally described as a *ringing in the ears*, but it may also take other forms such as buzzing, humming, clicking, or hissing. It has been reported by the American Tinnitus Association that approximately 15% of the general public, >50 million people in the United States, experience some forms of tinnitus, with 20 million people struggling with burdensome chronic tinnitus and >2 million cases characterized as extreme and debilitating [2]. A recent large research study conducted by Stohler et al [3] revealed an increasing incidence rate of tinnitus between 2000 and 2016. It was reported that the number of people living with chronic tinnitus is set to increase by more than half a million over the next decade, emphasizing a potentially increasing burden on the health care system. A recent observation is that the challenges associated with responses to the COVID-19 pandemic can increase tinnitus distress in case the people perceive the situation as generally stressful with increasing grief, frustration, stress, and nervousness [4]. It has been proved in previous studies that the presence of stress is highly correlated with tinnitus either initiating or worsening [5,6]. In fact, the British Tinnitus Association has reported a rapid increase in the number of people accessing their services, with a 256% increase in the number of web chats from May 2020 to December 2020 compared with the same period in 2019 [7].

### Tinnitus and Cognitive Impairments

Although most patients with tinnitus can cope well with the condition, managing to minimize its impact on their life, approximately 20% of the individuals can be characterized as being severely debilitated by their symptoms [8,9]. Recently, it has been proposed to differentiate between *tinnitus* to describe the auditory phantom percept and *tinnitus disorder* for the description of the auditory component plus the associated experience [10]. Although neuroimaging evidence is emerging showing that tinnitus is associated with abnormal functioning of the central auditory system [9,11,12], epidemiological studies have revealed that the perceived sound typically associated with the condition is not the only symptom. This suggests that other pathological elements may be associated with the condition; for example, the experience of tinnitus is related to a significant decline in cognitive functions such as working memory and attention [13,14], learning and learning rate [15], and cognitive speed [16], leading to an obvious decrease in quality of life. This involvement of nonauditory impairment is reflected by the fact that tinnitus is related to abnormal functioning not only in auditory brain areas but also in nonauditory brain areas, especially the prefrontal cortex [17], which plays a crucial role in executive control and decision-making [18]. Earlier studies that investigated cognitive impairments caused by tinnitus were mainly in the domains of attentional process and memory bias, and the findings were largely based on patients’ self-report behavioral and emotional responses to neuropsychological tests [15,19]. Andersson et al [20,21] were among the first to adopt experimental techniques from cognitive psychology, that is, the Stroop test, to measure selective attention in this context. Subsequent studies using similar methodologies further corroborated their findings that tinnitus depletes attention resources and results in compromised cognitive performance [16,20,22].

### Computational Modeling to Capture Cognitive Processes in Decision-making

Although behavioral summary statistics used in previous experimental cognitive studies, for example, accuracy and reaction time on the Stroop test, are more objective than self-report measures, they cannot be used to understand the underlying cognitive mechanisms that generate individual-level behaviors [23]. Computational modeling presents an alternative approach to make better sense of behavioral data and enhance our understanding of the cognitive processes in people with tinnitus. The most popular and successful application of computational modeling is in the field of learning and decision-making [24], which, surprisingly, has not been explored to date in the context of tinnitus.

Decision-making is a complex mental process that requires the coordination of several simultaneous cognitive processes, including perception, attention, evidence accumulation, and motor response networks [25,26]. It now seems that the cognitive abilities involved before (eg, perception and attention) or after (eg, learning) a choice is made can have significant influence on the final step of a motor response [27]; for example, attention is beneficial for decision-making because relevant features of the environment can be preferentially processed to enhance the quality of evidence. Executive functions and memory are critical to decision-making performance under risk [28].

Thus, we hypothesize that degradation in the cognitive abilities of patients with tinnitus may affect their decision-making characteristics. To gain a better understanding of the underlying cognitive processes of people with tinnitus, the method of computational modeling will be applied on a reward-loss version of the probabilistic decision-making under volatility task. This is a task that has been used to examine how humans can track the statistics of a reward-loss environment and adapt their learning rates accordingly [29]. Participants in their study had to choose 1 of 2 shaped Gabor patches, either of which might result in the delivery of an electrical shock. In each shape, a digital number is presented indicating the magnitude of the electrical shock that might be received. In the stable task block, 1 of the 2 shapes is associated with a 75% probability of receiving an electrical shock, and the other shape generated an electrical shock on the remaining trials. In the volatile task block, the shape most predictive of shock delivery reversed across several blocks of trials. We developed an equivalent web-based version of the task using the leprechaun story, which will be introduced in the Methods section. The computational parameters of this task can be used as a succinct representation not only for quantifying the effect of tinnitus on decision-making but also for investigating individual differences and within-individual changes in decision-making that are difficult to establish through superficial summary statistics.

### The Relationship Among Tinnitus, Cognition, and Psychological Disorders

It has been documented that, apart from impairments in cognition, patients with tinnitus may experience a variety of psychological disorders such as depression and anxiety. Holgers et al [22] found that the occurrence of depression and anxiety among a population consisting of people with severe tinnitus was significantly higher than that among the general population. Similar results were obtained in the study by Fetoni et al [30], where subjective tinnitus severity demonstrated a strong correlation with psychological distress measured by the Hospital Anxiety and Depression Scale. As a result, the identification of depression and anxiety disorders is of the highest importance in the management of patients with tinnitus because these comorbidities should be specifically treated [31].

It is widely recognized that the relationships between tinnitus and psychological variables are complex; for example, it is unknown whether cognitive impairments are caused by severe tinnitus directly or whether psychological factors are also involved, given that there is growing evidence for cognitive dysfunction among people with depression and anxiety [32,33]. In other words, cognitive impairments among people with tinnitus may not simply be the result of tinnitus but the co-occurrence or mediation of high levels of anxiety and depression [34]. Alternatively, tinnitus may lead to anxiety and emotional distress that, in turn, disrupt cognitive processes. Understanding the origin and underlying mechanisms of tinnitus and tinnitus-related impairment is therefore a significant challenge for current basic research. Psychological factors as well as impairments in cognition have been considered covariates to predict self-reported tinnitus severity. Interestingly, although both were identified as significant predictors of tinnitus severity, the decline in cognition has not been explained so far by psychological covariates in people with tinnitus [20,22]. However, this is not consistent with the literature in the field of anxiety and depression where both of these disorders can lead to cognitive impairments [33,35]. In addition, it was documented that these 2 psychological disorders can lead to reduced performance in decision-making tasks [36,37]; for example, it has been reported in the study by Browning et al [29] that individuals with high trait anxiety demonstrate less ability to adjust learning rates between stable and volatile environments in a laboratory-based reward-loss probabilistic decision-making task. We developed a web-based version of this task to examine whether it is tinnitus or a psychological alteration that is related to impaired decision-making performance in people with tinnitus.

### Take Tinnitus Severity Fluctuation Into Account

Another challenge encountered in the field of tinnitus research is that the perception of tinnitus loudness and distress is not constant in most cases but varies over time [38]. As a result, it is unknown whether the previous between-participants findings capture trait-like features of tinnitus or state-like features associated with fluctuating tinnitus symptoms. The second question of this study then relates to how moment-to-moment changes in tinnitus symptom experience affect decision-making performance under uncertainty. This question will be resolved by a longitudinal within-participants ecological momentary assessment (EMA) in which participants’ tinnitus states in the current moment will be sampled multiple times per day via self-report questionnaires, and the decision-making task will be triggered in several sessions based on their tinnitus severity. In contrast to retrospective self-report measures where patients are required to recall and summarize their tinnitus experience in the past 1 or 2 weeks, an EMA focuses on the current moment, minimizing the potential for recall bias and increasing ecological validity. The use of EMA in tinnitus studies has increased with the development of mobile apps and the growing availability of smartphones [39-41]. We have developed a mobile app, AthenaCX [42], that can automatically send notifications to the participants at several time points during the day requesting that they complete a state questionnaire asking about their current tinnitus symptom levels.

Furthermore, an intelligent algorithm is embedded in the app to trigger the decision-making task whenever the participants perceive relatively lower or higher levels of tinnitus distress than normal for them. Leveraging these tools, we are able to examine the dynamic changes of the computational markers extracted from decision-making behaviors associated with tinnitus fluctuation, simultaneously accounting for other time-varying factors—for example, emotions and nonadherence.

### Hypothesis

In summary, our first hypothesis is that people with chronic tinnitus demonstrate inferior learning adaptation in decision-making, represented by model-derived parameters, in a simulated uncertain environment compared with healthy controls, and this association is mediated by psychological disorders. An initial baseline cross-sectional study will be conducted to examine this hypothesis. Our second hypothesis is that the learning adapting ability for healthy controls will show good test-retest reliability, whereas the impairment-level of learning adapting of patients with tinnitus will exhibit higher variability and is positively correlated with moment-to-moment tinnitus severity in the longitudinal study.

## Methods

### Study Design

Once the participant is recruited, we will give them a unique ID and a link to download the study app AthenaCX from the Google Play Store or Apple Store. Participants will use the assigned ID to enter into the study app. After opening the app, they will be directed to read the plain language statement and the data privacy statement, which is referred to as participant information in the app. Once they agree to the terms, they will be asked to consent to the study. All communication with interested participants will be carried out on the web. The contact information will be hashed and saved on a secure password-protected Dublin City University Google Drive folder. All assessments will be completed inside the mobile app, except for the web-based decision-making task. The collected data will be stored in the AthenaCX databases, which reside on the Amazon Web Service platform at its Western Europe data center.

The experiment starts when the participant logs in to the app and consents to participate in the study. The complete study comprises 2 phases of experiments—the cross-sectional experiment and the longitudinal experiment—lasting up to 1 month depending on the frequency of patient responses. The cross-sectional experiment takes place right after the participants log in to the app and consent to participate. In this experiment, the participants are required to finish several baseline assessments, including the European School for Interdisciplinary Tinnitus Research Screening Questionnaire [43] (ESIT-SQ; part A for participants with tinnitus as well as healthy controls and part B only for participants with tinnitus) for an evaluation of their tinnitus-related history, the State-Trait Anxiety Inventory (STAI) [44] and Major Depression Inventory (MDI) [45] for both groups to score their anxiety and depression levels, and the Mini Tinnitus Questionnaire (MTQ) [46] specifically for participants with chronic tinnitus to assess their baseline tinnitus severity, followed by an EMA questionnaire asking about their current tinnitus symptoms and emotional status. In summary, the healthy controls will finish 3 questionnaires and a state questionnaire taking approximately 15 minutes, whereas the participants with chronic tinnitus will complete the same questionnaires as well as the tinnitus-specific questions in the ESIT-SQ and an extra baseline tinnitus assessment requiring ≤10 minutes. After finishing the questionnaires, they will be directed to the web-based reward-loss version of the probabilistic decision-making task, which requires 15 minutes to finish. The self-report questionnaires and decision-making task are introduced in detail in the sections that follow.

**Figure 1:**
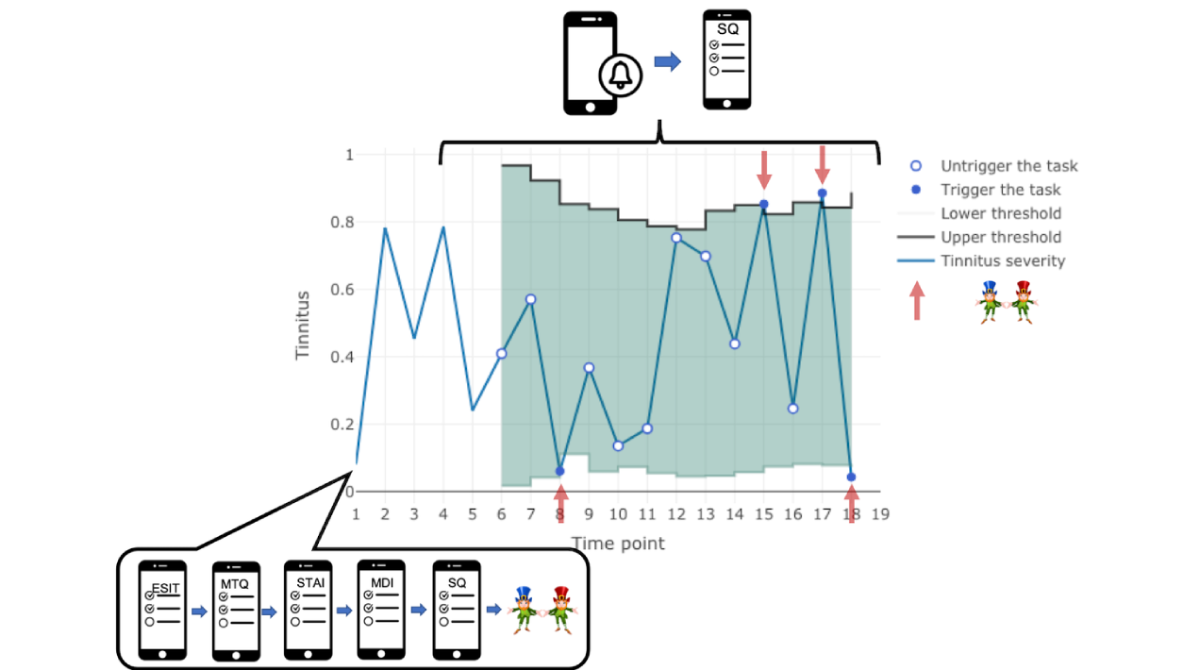
Pipeline of the experiments for tinnitus participants. ESIT: European School for Interdisciplinary Tinnitus Research Screening Questionnaire; MDI: Major Depression Inventory; MTQ: Mini Tinnitus Questionnaire; SQ: Screening Questionnaire; STAI: State-Trait Anxiety Inventory.

From the second day of the participants’ participation, the longitudinal-phase experiments will be activated. In the first part of this experiment, we will only observe the fluctuations of tinnitus perception and emotional status of both groups. This will be carried out as follows. The AthenaCX mobile app will automatically present to the participants the same EMA questionnaire that they completed in the baseline experiment. It is presented from 8 AM to 8 PM up to 4 times per day and is valid for 90 minutes. Unlike in the baseline experiment, the participants will not be directed to the decision-making task after the EMA survey until we obtain >5 responses of self-report tinnitus symptoms. From the sixth response, which is also the second part of this experiment, the algorithm we designed to intelligently trigger the decision-making task based on the participants’ current tinnitus symptoms will be activated. In other words, the algorithm will start monitoring the tinnitus severity reported by the participants with chronic tinnitus; only when the lower or higher thresholds (which are calculated for each participant combining all their tinnitus history reports) are reached will the decision-making task be triggered. Altogether, we expect the participants with chronic tinnitus to complete the decision-making task 4 times at 4 different time points: twice when their moment tinnitus symptom is smaller than the lower threshold and twice when their moment tinnitus symptom is larger than the higher threshold. Thus, the experiment will be terminated whenever the task is completed 4 times. Another termination condition is a time limit, that is, the experiment will come to an end after 1 month irrespective of the amount of data collected from the participant. The healthy controls will need to answer the same EMA questions, except for the tinnitus-related questions, with the same frequency as the patients with tinnitus for 2 weeks, during which the task will be triggered randomly 4 times. Refer to Figure 1 for the pipeline of the experiments for the participants with tinnitus.

### Study App (AthenaCX)

The study app AthenaCX is available for both Android and iPhone users. It is designed for the rapid creation and distribution of dynamic research surveys, including integrated consenting and even wearable data collection [47]. All of the surveys in this study will be delivered to the participants in this app. The demographic survey will be activated at the point of the initial download, whereas the other surveys will be activated through episodic triggering afterward; thus, only when the previous survey is completed will the next one be activated.

### The ESIT-SQ Measure

The ESIT-SQ [43] is a self-report tinnitus-relevant history questionnaire, which includes 39 multiple-choice questions. It is structured in 2 parts. Part A consists of 17 questions that can be used by both individuals with tinnitus and healthy controls. Seven questions require the participant to provide details of demographics, body characteristics, education, and lifestyle. One question is about family history, and 9 questions ask medical history and presence of hearing-related and other symptoms. The last of these questions screens for presence of tinnitus lasting for >5 minutes over the past year. Participants who answer *yes* to this question will be directed to complete the 22 questions in part B, which includes 8 questions about tinnitus perceptual characteristics, 1 general question about the impact of tinnitus, 6 questions about onset-related characteristics, 4 questions about tinnitus modulating factors and associations with coexisting conditions, 1 question on objective tinnitus, and 2 health care–related questions.

### The MTQ Measure

The MTQ [46] is the short version of the Tinnitus Questionnaire used to examine subjective distress related to tinnitus. It consists of 12 questions reflecting the most pertinent aspects of tinnitus distress with 3 potential answers: *true, partly true,* and *not true*, each yielding a score from 0 to 2. The MTQ has shown good test-retest reliability as well as high validity. The MTQ score will serve as the primary outcome measure for tinnitus severity in this study.

### The STAI Measure

The STAI [44] is a commonly used measure that includes two 20-item self-report scales for assessing trait and state anxiety. *State anxiety* refers to the current feeling of the respondent, whereas *trait anxiety* refers to the general feeling of the respondent. Items on the state scale are rated on a 4-point scale from *not at all* to *very much so*; items on the trait scale are also rated on a 4-point scale, but here, the ordinal labels range from *almost never* to *almost always*. The total score of each scale ranges from 20 to 80, with higher scores indicating greater anxiety. Scores ≥30 indicate moderate anxiety, and scores ≥45 indicate severe anxiety [48]. Internal consistency coefficients for the scale range from 0.86 to 0.95. Test-retest reliability of this measure ranged from 0.65 to 0.75 over a 2-month interval [44].

### The MDI Measure

The MDI [45] is a 12-item self-report measure for depression developed by the World Health Organization’s Collaborating Center in Mental Health. Items contained in the MDI reflect all symptoms of depression in the Diagnostic and Statistical Manual of Mental Disorders, Fourth Edition, and the International Classification of Diseases, Tenth Revision. Each item is rated on a 6-point scale from *at no time* to *all the time* to assess the presence of a depressive disorder and the severity of depressive symptoms over the past 2 weeks. The reliability of the MDI as a measure of depression severity is 0.89 based on the results in the study by Cuijpers et al [49]. Scores on both the STAI and the MDI will be used as covariates along with tinnitus severity to examine the contribution of self-reported anxiety and depression to the performance on the cognitive decision-making task.

### The EMA Survey of Tinnitus Symptoms and Emotional Status

**Figure 2:**
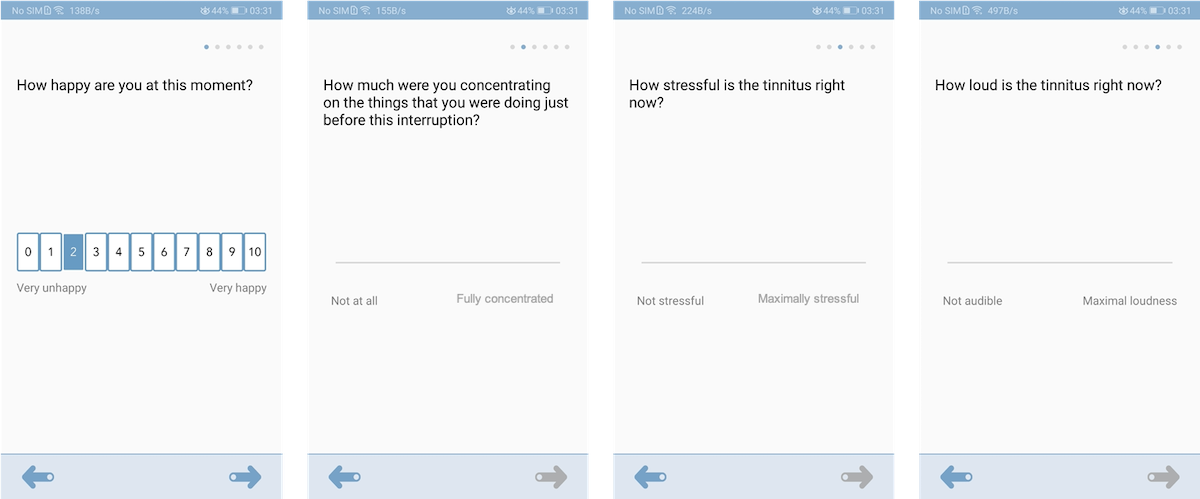
Screenshots of the interfaces of the ecological momentary assessment questions on the smartphone screen.

An EMA approach is used to allow in-the-moment responses from participants. Typically, the EMA survey takes <1 minute to complete. It consists of 4 questions. The first question asks the participant to rate their emotional valence on a scale ranging from 0 to 10, representing *Very unhappy* to *Very happy*. The second question asks how much they were concentrating on the things that they were doing before the interruption. The participants can provide their answers on a visual analog scale (VAS) by moving a slider between the end points from *Not at all* to *Fully concentrated*. Technically, the VAS is implemented as a slider without a preset initial position to avoid anchoring affects. The last 2 questions are specifically for patients with tinnitus and ask about tinnitus loudness and tinnitus stressfulness. These questions are also answered using a VAS. The anchor points of the VAS asking about tinnitus loudness are *Not audible* at one end and *Maximal loudness* at the other. For the VAS asking about tinnitus stressfulness, the labels are *Not stressful* on the left side and *Maximally stressful* on the right side. In Figure 2, we have provided the interfaces of the EMA questions on the smartphone screen as an example to demonstrate the implementations of the surveys on the AthenaCX app.

### The Triggering Algorithm

The decision-making tasks are triggered by the triggering algorithm developed in the study by Monacelli et al [50]. In our context, we aim to balance the burden placed on the participants—do not trigger the task too often—and data quality—collect enough data for the analysis (refer to the Statistical Analysis section). Monacelli et al [50] have shown that their algorithm performs better than both a random schedule and a rule-based approach with prefixed thresholds, which are the current state-of-the-art approaches, in achieving this goal. The algorithm adapts to the individual participants based on their reported history of tinnitus severity and adherence. It relies on 2 statistical models: tinnitus severity is modeled as an independent and identically distributed sample from a beta distribution and adherence as an independent and identically distributed sample from a Bernoulli distribution. At each interaction of the participants with the app, the algorithm estimates both the parameters of the beta distribution and the adherence rate. By using the estimates of the beta distribution and a control chart approach, the algorithm defines adaptive CIs for the tinnitus severity, which are used as adaptive thresholds for triggering the decision-making task. The significance level of these CIs is chosen by analytically solving a design optimization problem. This problem, in particular, formalizes the intent of the authors to trigger on average a prefixed number of additional tasks per participant, thus balancing the burden placed on the participants and data quality. The result is a closed-form solution for the significance level that depends only on the length of the experiment and the first time point after which the decision-making tasks can be triggered. Finally, by considering the estimated adherence rate, the algorithm updates the optimal significance level by replacing the length of the experiment with an estimate of the final number of samples collected for the individual participant. This results in an algorithm that is more careful in submitting the task to adherent participants but tries to collect data as soon as possible for those who are less adherent. This process is repeated for each interaction of the participant with the app, resulting in a web-based adaptive algorithm. A representation of the algorithm is presented in Figure 1.

As suggested in the study by Monacelli et al [50], this procedure is activated once participants have provided data via the app a minimum of 5 times, and we stop the algorithm after 5 triggers. We anticipate, based on past adherence behavior, that approximately 35% of the participant cohort will interact with the app >5 times [50]. A data quality assessment for the triggering algorithm will be performed by repeating the analysis conducted in the study by Monacelli et al [50]. Therefore, we will consider both the *F*_1_-scores and the utility measures, which represent, respectively, the precision of the algorithm and its effectiveness in balancing data quality and the burden placed on the participants. Plots of both the empirical cumulative distribution function and a Mann-Whitney-Wilcoxon test will be considered. It should be noted that although the triggering algorithm accounts for the uncertainty of the sample size and incorporates it into its definition of high and low values, this uncertainty cannot be overlooked in the analysis (refer to the Statistical Analysis section). Therefore, a scatterplot of the total number of samples against variables of interest such as the learning rate of the participants will be considered.

### The Decision-making Task

**Figure 3:**
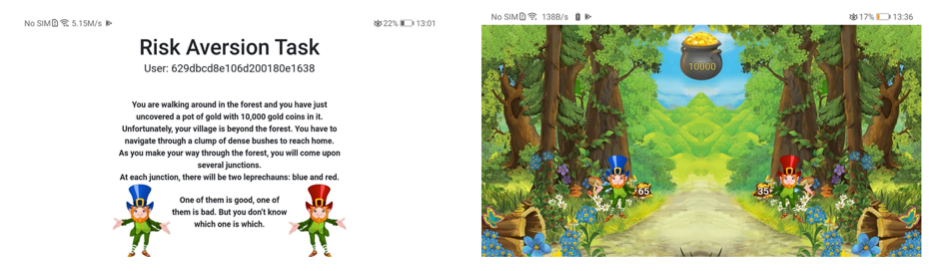
Screenshots of the web-based leprechaun task.

**Figure 4:**
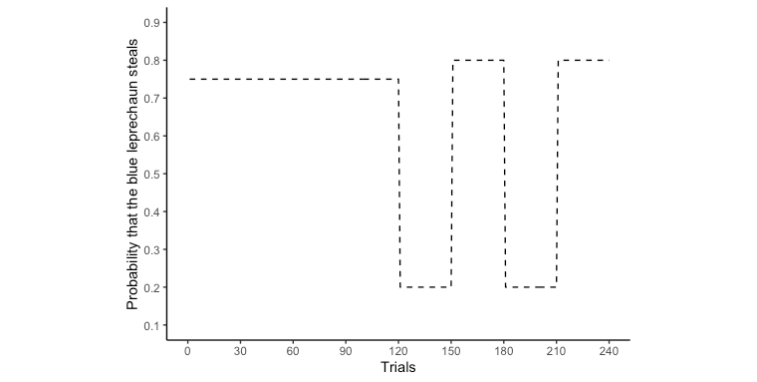
An example of the changes in the steal probability of the blue leprechaun.

Performance on the decision-making task is captured as an objective measure of adaptation of learning rate to volatility. In the task, participants are told that they are walking through a forest carrying 10,000 gold coins (refer to Figure 3 for screenshots of the task). As they proceed through the forest, they will come across a series of junctions. At each junction, there will be 2 leprechauns, each with distinct behaviors (therefore, we may also refer to this task as the leprechaun task). The leprechaun wearing a blue hat is referred to as the blue leprechaun, and the leprechaun wearing a red hat is referred to as the red leprechaun. The participants have to choose between the blue and red leprechauns to pass through each junction. They should choose carefully because one of the leprechauns will steal gold coins from them and run away. One of the leprechauns has a high probability of stealing gold coins from the participants, whereas the other one has a low probability. However, the probabilities of stealing gold coins, or, as we refer to them, the action-outcome contingences of the leprechauns, can be altered as part of the experimental design. In this particular experiment we have 2 blocks. In the stable block of 120 trials, the probability of one of the leprechauns stealing is consistently 75%, whereas the probability for the other leprechaun is consistently 25%. In the volatile block, which also comprises 120 trials, the stealing probability switches between 80% blue leprechaun and 80% red leprechaun every 30 trials. Figure 4 shows an example of the change of the probability of theft by the blue leprechaun throughout the task. As the participants are required to perform this task multiple times, the action-outcome contingencies of the 2 leprechauns are reversed each time the task is activated to eliminate memory effects, that is, if the structure of the task remains fixed throughout the experiments, the more times the participants play, the better they perform because they gain more experience and will be able to remember the *correct answers*.

It is not known to the participants that the task consists of 2 blocks and 120 trials (each trial is an opportunity for making a choice in the game in which the participants choose between the 2 leprechauns) per block. Each leprechaun holds a bag in their hands with a number on it that represents how many gold coins the participants will lose if that leprechaun steals from them. Throughout the task, the potential losses for choosing one of the leprechauns are randomly generated between 1 and 100 (*M_1_*), and the losses for the other leprechaun are set to 100 – *M_1_*.

Participants will be instructed that they should base their assessment of the most trustworthy leprechaun on their recent outcome history with each leprechaun and that their goal is to get back to their village with as many gold coins as possible. They need to learn which leprechaun on average is currently the best one to choose throughout the task, and they also need to adjust the speed of learning to reflect the stability or volatility of the environment.

### Ethics Approval

This study has been approved by the Dublin City University research ethics committee (DCUREC/2021/070). People who are interested in participating will be instructed to access the study app (AthenaCX) where the plain language statement and consent form are presented to the participants before entering into the study. They will only be able to proceed if they provide consent.

### Recruitment

Two groups, that is, patients with tinnitus and healthy controls, will be recruited in this study, and we will limit our recruitment to Ireland. Power analysis was performed to determine the sample size required to obtain significant results. The significance of the between-group difference and the correlation between adaptive learning ability and tinnitus symptoms are considered, and it turns out that the minimum sample size for satisfying both analyses is 54 with medium effect size. Thus, we will recruit 60 patients with tinnitus and 60 healthy controls. The 2 groups will be recruited through separate advertising campaigns, and both will be paid for their participation. To incentivize the participants to be adherent with the requests for data as well as to engage with the experimental decision-making task to the best of their ability, the payment is a One4all gift card; the value of the card is composed of two parts, that is, there is a basic payment (€10 [US $9.86]) plus a variable bonus (up to €30 [US $29.57]). The bonus will be determined by their response rates and performance in the decision-making task (measured by the gold coins remaining in each session) in the experiments. If the response rate to the EMA surveys is >50%, the participant is eligible to earn a bonus of €10 (US $9.86). The participants need to complete the decision-making task 4 times, each worth up to €5 (US $4.93), which means they will get up to €20 (US $19.72) for completing the decision-making task.

The patients with chronic tinnitus will be recruited through clinics and tinnitus support groups and associations; where possible, measures of hearing loss will be captured. We have established connections with The National Charity for Deafness and Hearing Loss and audiologists from Otologie Tinnitus Care (a tinnitus clinic in Dublin, Ireland). Both will help with the recruitment of patients with tinnitus. We will use an advertisement seeking people whose lives are affected by tinnitus. Eligible participants for the group of patients with chronic tinnitus will be those aged between 18 and 70 years who have experienced subjective tinnitus for ≥6 months and have access to a smartphone with internet capability. The healthy group will be recruited through our clinical partners and by posting advertisements on social media. The same doctors helping recruit patients with tinnitus will also be asked to identify likely healthy matches for the patients with tinnitus that they recruit. Thus, we will recruit the group of patients with tinnitus first, which will allow us to establish a distribution of age and gender that we will match in the subsequent round of healthy control recruitment. The healthy participants will be matched with the patients with tinnitus of the same gender and similar age with a difference of up to 5 years. Healthy participants who are interested in participating in the experiment can email us and will be recruited if they suit our study-matching needs.

**Figure 5:**
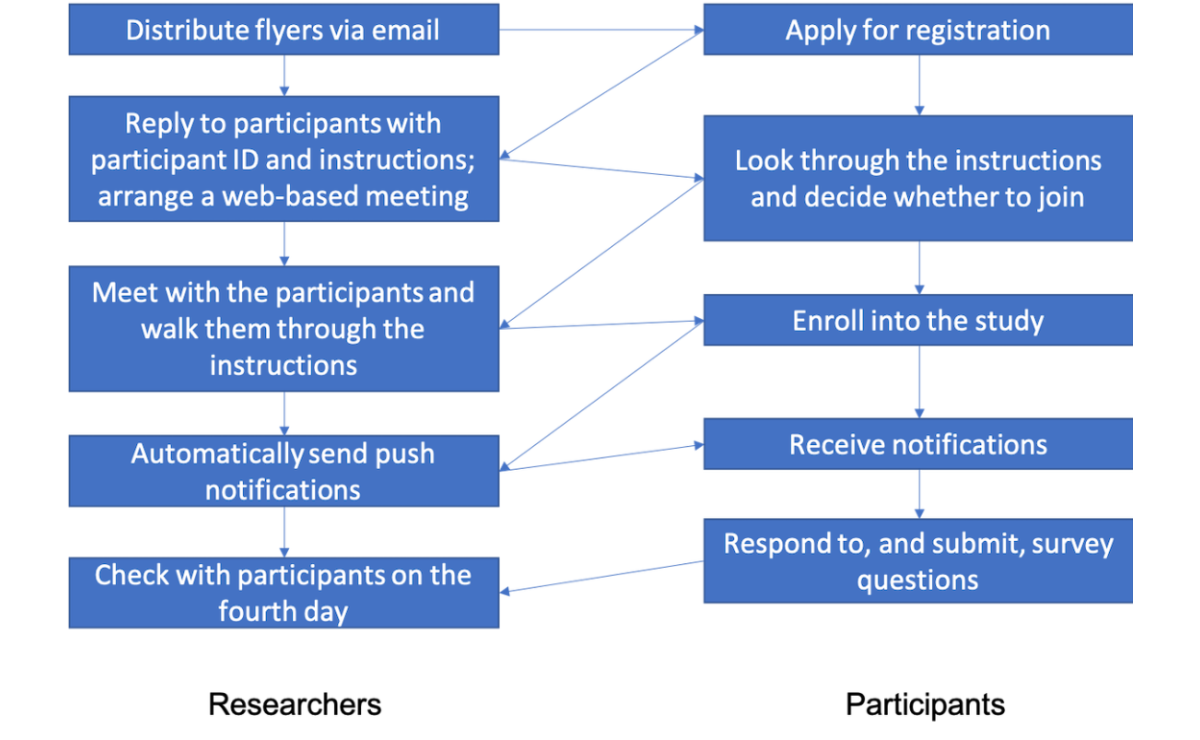
The workflow of the recruitment process.

All participants will need to register their interest via the link provided in the recruitment poster. We will get back to them as soon as we receive their registration information. We will forward the instructions for joining the experiment and ask each participant to meet one of the investigators on the web in case they have any questions. This step alone, while adding to the time burden for the researcher, should improve data quality and reduce the chances of multiple enrollments by the same person. We will also follow up with the participants on the fourth day to check whether they had any issues with receiving notifications and to encourage them to be more positively engaged. Please refer to Figure 5 for the workflow of the recruitment process.

### Statistical Analysis

#### Baseline Self-report Analysis

Standard descriptive statistics will be applied to describe the baseline assessments of the 2 groups, including demographic characteristics, tinnitus symptom severity, and psychological assessments. Continuous variables will be summarized by measures of central tendency and variability, whereas categorical variables will be described by measures of frequency and relative frequency. Correspondingly, a 2-tailed *t* test will be used for assessing differences in the distributions of continuous variables between the 2 groups, and the Pearson chi-square test will be applied to categorical variables.

#### Baseline Behavior Data Analysis

##### Overview

The baseline behavior data collected from the volatility decision-making task will be analyzed on three levels—basic exploratory data analysis, nongenerative computational modeling analysis, and generative computational modeling analysis—to capture the differences in decision-making under contingency volatility between the healthy controls and patients with tinnitus. The nongenerative analysis will investigate the effects of loss and the magnitude of loss on the choices during the task, whereas the generative analysis will reveal the underlying cognitive processes while participants make decisions. The details of the 3 levels are presented in the following sections.

##### Exploratory Data Analysis

We will first conduct an exploratory analysis of the behavioral choice data collected in the baseline experiments to capture the choice preferences of the patients with chronic tinnitus and the healthy controls. Several model-independent measures of behavior will be considered; for example, we will visualize how often participants choose the good leprechaun, that is, the leprechaun less likely to steal gold coins (ie, percentage of minimizing probability of potential losses) and how often they choose the leprechaun with the smaller stealing magnitude (ie, percentage of minimizing magnitude of potential losses). We will also explore the probability of repeating and switching an action, that is, win-stay and lose-shift, to capture fundamental aspects of learning. All of these measures will be visualized at 3 levels. At the trial level (averaging across participants), the plot demonstrates the trial-by-trial dynamic of the choice behavior. At the participant level (averaging across trials), the plot illustrates individual variation. At the overall level (averaging across both trials and participants), the plot provides the average performance of the whole group.

##### Nongenerative Computational Modeling Analysis

A logistic regression model will be trained to predict participants’ learning dynamics. It will estimate the probability of staying versus switching based on the loss in the previous trial (main effect of the loss), current difference in loss magnitudes between the 2 options (main effect of potential loss magnitudes), and the interaction (loss × difference in loss magnitudes).

##### Generative Computational Modeling Analysis

To understand the underlying decision-making processes, generative computational models are developed to break performance down into several interpretable cognitive components. Several computational models have been developed and are described in the literature [29,51]. The common feature of these models is that they all include a learning rate parameter for capturing the extent to which the outcome probability estimates are adjusted, given the unexpectedness of the previous trial’s outcome; a risk preference parameter to allow for individual differences in how they weight outcome magnitude versus outcome probability; and an inverse temperature parameter to control the degree to which the expected values are used in determining the option chosen. What we refer to here as *model 1* and *model 2* in the study by Gagne et al [51] are introduced for demonstration purposes. It is worth noting here that various models will be fitted, except for these 2 models, and model comparison will be conducted to select the model that best describes the behavioral data, subject to appropriate validation processes to avoid overfitting.

*Model 1* supposes that the probability *P_t_* that a good outcome (no loss of coins) would result from choosing the blue leprechaun rather than the red leprechaun is updated on a trial-by-trial basis using the Rescorla-Wagner rule.

*P_t_* = *P_t_*_–1_ + *α*(*O_t_*_–1_ – *P_t_*_–1_)

in which the learning rate *α ∈* (0, 1) determines how much weight the decision-maker gives to the recent outcomes when updating their expected probability. The outcome *O_t_*_–1_ is coded as 1 if the blue leprechaun is chosen and produces a good result or if the red leprechaun is chosen, followed by a bad result. *O_t_*_–1_ is coded as 0 for the opposite situation in the task. The initial outcome probability *P_t_* is set as 0.5.

The outcome probability estimate is then adjusted to *P_t_'* using a risk preference parameter (*γϵ*(0, 10)) to capture the relative importance of the magnitude of losses versus the outcome probability. If *γ*<1, it means that the participant places greater weight on the magnitude of losses, whereas if *γ*>1, it means that the participant places greater weight on outcome probability when performing a choice.

*P_t_'* = min {max[(*γ*(*P_t_* – 0.5) + 0.5), 0], 1}

The expected value for each leprechaun is then calculated through multiplying the adjusted outcome probability and loss magnitude separately, before taking the difference in expected values between the 2 leprechauns.

*v_t_* = *P_t_'M_t_^blue^* – (1 – *P_t_')M_t_^red^*

Finally, the action probabilities are generated using a softmax function with an inverse temperature parameter *β*, which controls the degree to which the expected values are used in choosing the leprechauns.



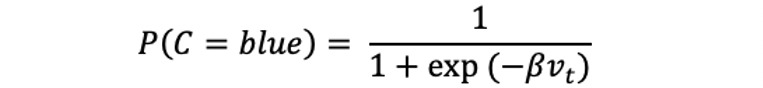



*Model 2* uses the same assumption as *model 1* in terms of updating the outcome probability (Rescorla-Wagner rule). However, in contrast with *model* *1*, *model* *2* assumes that the decision-makers combine outcome probability and outcome magnitude additively, using a mixture weight (*λ*). Furthermore, the difference in outcome magnitudes is nonlinearly scaled with a scaling parameter (*r* *∈* [0.1, 10]) to capture any potential bias that the participants have toward treating the differences in outcome magnitudes.

*v_t_ = λ*[*p_t_* – (1 – *p_t_*)] *+* (1 – *λ*)[*M_t_^blue^* – *M_t_^red^*]*^r^*

This model also incorporates a choice kernel (*k_t_*), which acts like an average window moving forward as the trials proceed. It is updated by an update rate parameter (*η ∈* (0, 1)), which can be used to determine the number of recent choices contained in the value of the choice kernel on the current trial.

*K_t_ = k_t_*_–1_*+ η*(*C_t_*_–1_ – *k_t_*_–1_)

The expected value and the choice kernel are both passed through a softmax function to decide the probability that the blue leprechaun is chosen in the current trial with two separate inverse temperatures (*β* and *β_k_*).



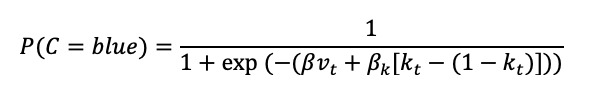



There are 3 free parameters (*α, γ*, and *β*) in *model 1* and 6 free parameters (*α, λ, r, η, β,* and *β_k_*) in *model 2*. All of the generative models will be estimated with the hierarchical Bayesian method, in which we assume that the parameters of individual participants are generated from parent distributions.

The primary measure of interest in the leprechaun task is whether the participants adapted their learning rates in response to the changes of the environment (from stable block to volatile block). To take the potential within-participant correlation of the stable and volatile parameters into account in the modeling process, we assume that each participant had a stable and volatile parameter with a prior distribution defined by the multivariate normal distribution with means *μ_stable_* and *μ_volatile_* and covariance matrix Σ, which can be converted to a correlation matrix. The model will be implemented in Stan [52], a probabilistic programming language, so that the parameters can be estimated using Markov chain Monte Carlo algorithms. The Cholesky decomposition trick has been widely used in the Monte Carlo method for simulating systems with multiple correlated variables [53]. Thus, in the implementation, we will use this technique to decompose the correlation matrix into the product of a lower triangular matrix (Cholesky factor) and its transpose; thus, the correlation matrix of the stable and volatile parameters can be derived.

##### The Impacts of Baseline Tinnitus Severity and Psychological Measures

The impacts of baseline self-reports, including tinnitus, anxiety, and depression, on participants’ adaptation performance on the task are then analyzed. We first extract the median values of each participant’s parameters and calculate the differences between the paired stable and volatile parameters estimated in generative computational modeling analysis. The differences of each participant are then used as the outcome variable in a linear regression analysis, with baseline tinnitus severity as well as anxiety and depression scores as predictors.

##### Longitudinal Data Analysis

Our next question concerns whether the computational markers of the contingency volatility decision-making task of the participants with tinnitus can be predictors for detecting moment-to-moment tinnitus symptom severity. The behavioral data of the 2 groups collected in the longitudinal experiment will be fitted to the best fitting model obtained in the baseline generative modeling analysis. Our primary analysis for the participants with tinnitus will be repeated measures linear regression, with the EMA tinnitus severity as outcome variable and the model-derived parameters that capture adaptation ability of learning as independent variables. This analysis estimates the level of tinnitus distress when a patient with tinnitus is more capable of learning the contingency volatility of the environment compared with when the same patient is less capable of learning the contingency volatility of the environment. In the second model, emotional status will be considered as another independent variable together with the model-derived parameters to predict tinnitus severity, that is, multiple regression, so that the impacts of emotional status can be examined. For the healthy controls, we will mainly focus on the test-retest reliability of their task performance.

## Results

The implementation of the experiments has been completed. We have tested the workflow of the experiment, the timing of the EMA surveys, the notification functionality on various Android and iOS systems, and so on. Everything works as anticipated. For the recruitment, we designed a poster in which a link is embedded so that participants can register their interest as soon as they read the advertisement. We also developed various versions of the instructions (text, interaction-enabled, and video versions) that demonstrate how to engage with the study step by step. All of these materials have been shared with the audiologists who have agreed to help with the recruitment. The data collection effort will take place over 12 months. We will start the analysis as soon as the data collection is finished. The results are expected to be published in December 2023.

## Discussion

### Overview

The hypothesis of this study is that patients with chronic tinnitus demonstrate impaired adaptive learning ability in changing environments compared with a healthy population. This impairment might be exacerbated at the moment when the patients are experiencing tinnitus symptoms. This is the first study to investigate the impacts of tinnitus on decision-making and the first to acquire computational markers to investigate what differences, if any, exist between the cognitive processes of patients with tinnitus and a healthy population. In addition, by leveraging smartphone technology, this study will be the first, to the best of our knowledge, to perform many repeated measures of decision-making based on real-time EMAs of tinnitus symptoms in a real-world population consisting of patients with tinnitus, making it uniquely possible (compared with cross-sectional designs and traditional methods) to capture ongoing tinnitus vulnerability.

The findings and implications of this study will be presented to the audiologists we are working with as well as the scientific community. Identifying objective measures robustly associated with tinnitus fluctuation is important for monitoring trajectories of tinnitus development as a result of potential treatment. If the fluctuation of tinnitus symptoms is truly associated with decision-making performance, the computational phenotypes extracted have the potential to serve as objective measurements of tinnitus severity in the future. The clinical management and treatment of patients with tinnitus will benefit from these potential computational markers.

### Limitations

Nevertheless, this study also includes limitations. Participants may fail to respond to the symptom survey request and therefore fail to accumulate the required number of EMA data points to feed the calculation that decides upon delivery of the decision-making task. Such neglect may arise because of competing tasks and priorities or interruptions that arise from the normal activities of daily living. The bonus payment feature has been introduced to address this issue and improve response rates. Another limitation is that it is possible that the decision-making task may never be triggered because of the probabilistic nature of the algorithm, although the triggering algorithm has been designed to overcome this limitation by adapting to individual adherence behavior.

## Abbreviations

EMA: ecological momentary assessment
ESIT-SQ: European School for Interdisciplinary Tinnitus Research Screening Questionnaire
MDI: Major Depression Inventory
MTQ: Mini Tinnitus Questionnaire
STAI: State-Trait Anxiety Inventory
VAS: visual analog scale

## Data Availability

The data sets generated and analyzed during this study will be anonymized and made available via open science at Zenodo.
